# Clinical significance of IgG deposition in the glomerular mesangial area in patients with IgA nephropathy

**DOI:** 10.1007/s10157-012-0660-0

**Published:** 2012-07-03

**Authors:** Yukihiro Wada, Hiroaki Ogata, Yui Takeshige, Akiko Takeshima, Noriyo Yoshida, Masahiro Yamamoto, Hidetoshi Ito, Eriko Kinugasa

**Affiliations:** Nephrology Department of Internal Medicine, Showa University Northern Yokohama Hospital, Chigasaki-chuo 35-1, Tsuzuki, Yokohama 224-8503 Japan

**Keywords:** IgA nephropathy, IgG deposition, IgA-IgG nephropathy

## Abstract

**Background:**

Immunoglobulin (Ig) A nephropathy (IgAN) is characterized by mesangial deposits of IgA1 and C3, often with co-deposits of IgG. We attempted to clarify the clinical significance of mesangial IgG deposition in patients with IgAN.

**Methods:**

We retrospectively reviewed 57 patients who were diagnosed with IgAN on the basis of pathological examination of renal biopsy specimens obtained between October 2006 and December 2010. Subjects were divided into two groups: IgA+IgG deposition (IgA-IgG) group (*n* = 29) and IgA deposition alone (IgA) group (*n* = 28). The study outcome was complete remission (CR), defined as negative proteinuria by dipstick urinalysis and urinary erythrocytes of less than 1–4/high-power field.

**Results:**

Proteinuria was greater in the IgA-IgG group than the IgA group (1.1 ± 0.8 vs. 0.7 ± 0.6 g/day, Mann–Whitney *U* test, *P* = 0.042). Capillary wall IgA deposits were noted more frequently in the IgA-IgG group than the IgA group (59 vs. 11 %, Fisher’s exact test, *P* = 0.014). During the median follow-up period of 33.3 months (range 6–55 months) in the 57 patients, we observed CR in 24 cases (42.1 %). After the start of treatment, urinary abnormalities disappeared earlier in the IgA group than in the IgA-IgG group (log rank test, *P* = 0.012). Cox’s regression model showed that IgG deposition reduced the hazard ratio for CR (hazard ratio 0.35; 95 % confidence interval 0.14–0.82, *P* = 0.014). Therefore, IgG deposition is a risk factor for persistent urinary abnormalities.

**Conclusion:**

Mesangial IgG deposition is associated with more severe clinical features in patients with IgAN.

## Introduction

Immunoglobulin (Ig) A nephropathy (IgAN) is the most common glomerulonephritis and is a principal cause of chronic kidney disease (CKD). Generally, it is known that Caucasians and Asians are more prone to IgAN than persons of African descent. In particular, southern Europe and parts of the Asia–Pacific region are known to have a high prevalence rate of IgAN [1]. In Japan, surveys of IgAN that have been undertaken since the early 1970s have shown that more than 30 % of adult patients and more than 20 % of children with chronic glomerulonephritis have this disease [2]. Mesangial cell proliferation and extracellular matrix expansion are found in patients with mild IgAN, while progressive glomerular and interstitial sclerosis in severe IgAN leads to endstage kidney disease (ESKD) in 30–40 % of patients within 20 years after diagnosis [3].

In 1968, IgAN was described by Berger et al. as “primary glomerulonephritis, exhibiting mesangial IgA and IgG co-deposition” [4]. IgAN is also characterized by mesangial deposits of IgA1 and C3, often with co-deposits of IgG [5–7]. However, since IgG deposits are not essential to the definitive diagnosis of IgAN, little attention has been paid to the clinical significance of IgG deposition. In addition, there are some unclarified issues on the difference between nephropathy characterized by deposits of both IgA and IgG (IgA-IgG nephropathy) and nephropathy characterized by deposits of IgA alone.

Therefore, we conducted this historical cohort study to assess whether mesangial IgG deposition is clinically significant in patients with IgAN.

## Patients and methods

### Study subjects

From October 2006 to December 2010, 63 patients were diagnosed as primary IgAN at Showa University Northern Yokohama Hospital. The diagnosis of IgAN was based on light microscopic findings of mesangial proliferative changes, immunofluorescence study findings of mesangial IgA and C3 deposition, and electron microscopy findings of electron-dense deposits in glomerular mesangial tissue obtained by renal biopsy. From a total of 63 patients, 6 patients inferred to have complication of other renal diseases such as Henoch–Schönlein purpura nephritis, diabetic nephropathy, benign nephrosclerosis, nephropathy of toxemia of pregnancy and hepatic IgAN were excluded. Finally, 57 patients, including 27 males and 30 females, aged 16–61 years (mean 37.1 years), were enrolled as the subjects of this study.

### Study design

This is a historical cohort study. The study protocol was approved by the Showa University Northern Yokohama Hospital Institutional Review Board. The observation period was from October 2006 to June 2011. Study patients were divided into two groups according to the presence of IgG deposition: IgA-IgG group, with both IgA and IgG deposits (*n* = 29), and IgA group, with IgA deposits alone (*n* = 28). The outcome assessed was complete remission (CR) of urinary abnormalities, which was defined as complete absence of proteinuria by dipstick analysis and urinary erythrocytes less than 1–4/high-power field (HPF).

### Assessment of IgG deposits

**Fig. 1:**
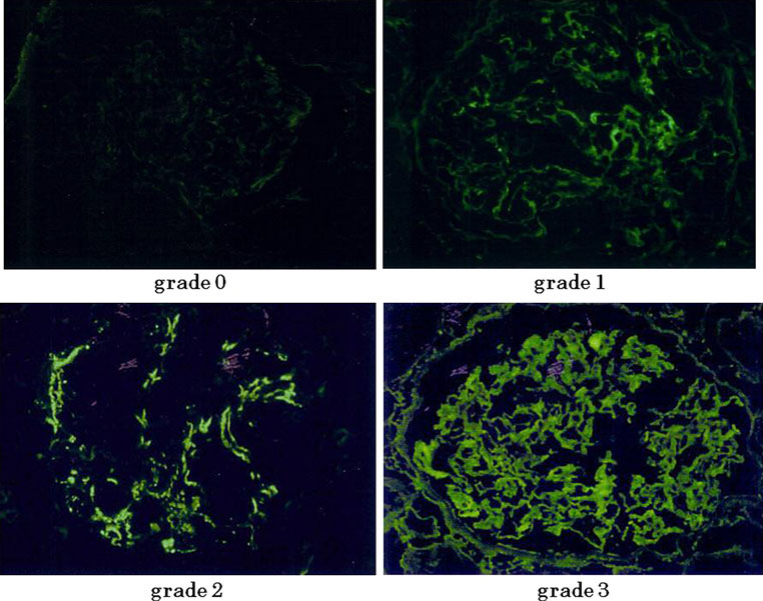
Assessment of IgG deposits. The extent of IgG deposition was graded on a 4-point scale: grade 0, absent; grade 1, weak; grade 2, moderate; grade 3, severe

**Fig. 2:**
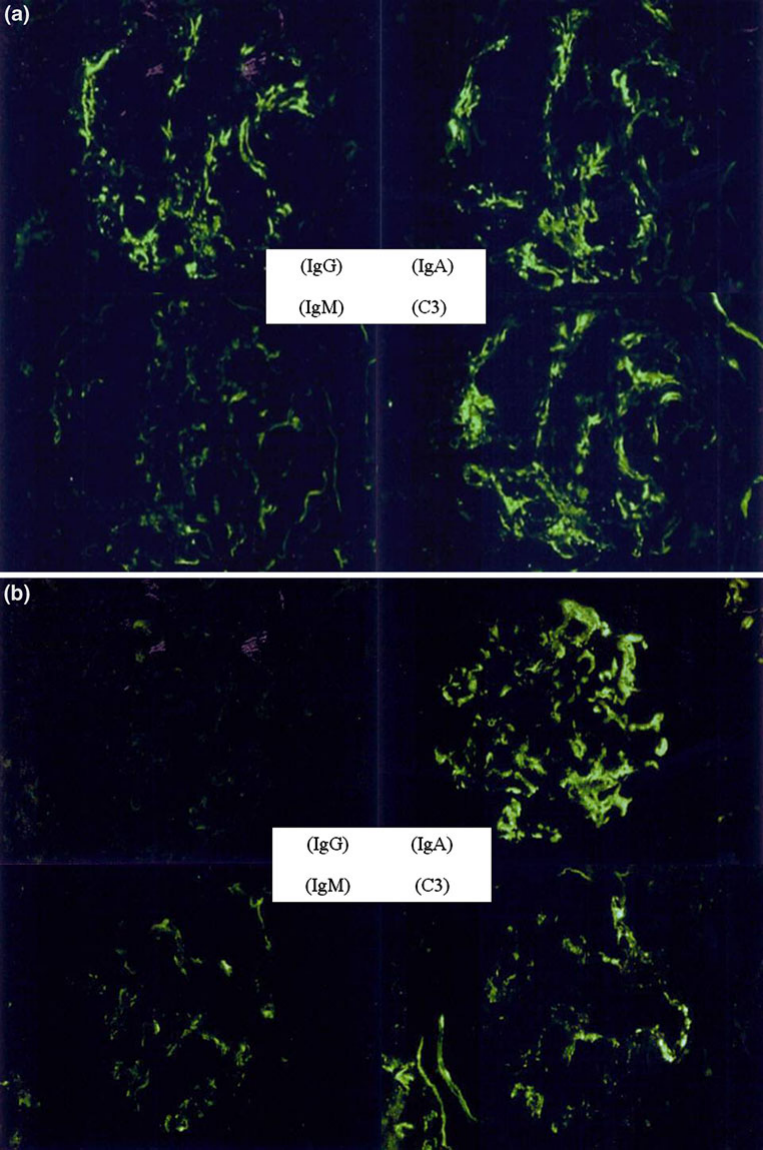
**a**, **b** Glomerular mesangial immunofluorescence findings (stained with fluorescein isothiocyanate-conjugated monospecific antisera against human IgG, IgA, IgM and C3c). **a** Typical images in the IgA-IgG group. **b** Typical images in the IgA group

Using immunofluorescence techniques, two pathologists independently examined the biopsied glomerular tissue for mesangial IgG deposits. In addition, two nephrologists with extensive experience in the diagnosis of IgAN and the two pathologists discussed and concluded when the two pathologists evaluated the mesangial IgG deposition differently. The extent of IgG deposition in the mesangial area was graded on a 4-point scale: grade 0, absent; grade 1, weak; grade 2, moderate; grade 3, severe (Fig. 1). Segmental mesangial deposit levels (grade 2 or higher) or global mesangial deposit levels (grade 1 or higher) were considered as indicative of IgG deposits (Fig. 2a, b), referring to the previous reports [8]. Furthermore, we paid attention not only to mesangial but also to capillary wall IgG deposits, and assessed the location of IgG deposits in the glomerulus of IgA-IgG group patients.

### Assessment of the immunostaining findings of IgA

We assessed immunostaining findings of IgA as well as IgG. Using a method similar to the assessment of IgG deposits, we examined the IgA deposits between the two groups. The immunofluorescence intensity of IgA in the mesangial area was graded on a 3-point scale: grade 1, weak; grade 2, moderate; grade 3, severe. Furthermore, we evaluated whether there was any difference in the location of glomerular IgA deposits between the two groups.

### Factors assessed

Baseline clinical characteristics, including information on follow-up period, duration from the onset of proteinuria to the time of renal biopsy (duration from onset), age, gender, body mass index (BMI), systolic and diastolic blood pressure, urinary findings, serum creatinine levels (sCr), estimated glomerular filtration rate (eGFR) and therapeutic regimens, were obtained from the patients’ records. Blood pressure of 130/80 mmHg or higher was defined as hypertension. Hematuria was assigned one of the following scores: 0, absent or 1–4 red blood cells (RBCs)/HPF; 1+, 5–9 RBCs/HPF; 2+, 10–19 RBCs/HPF; 3+, more than 20 RBCs/HPF. eGFR was calculated by the modified Modification of Diet in Renal Disease (MDRD) equation for Japanese people [9]. Therapeutic regimens were analyzed in each group, including information on the use of renin–angiotensin system inhibitors (RASI), antiplatelet drugs, oral steroids and immunosuppressants. In addition, use of tonsillectomy, pulsed steroid therapy and pulse steroid therapy combined with tonsillectomy (ST) were also analyzed in each group. The indication for tonsillectomy was otolaryngological findings of chronic tonsillitis with hypertrophic or atrophic tonsils with inflammatory debris in the tonsillar crypts. The regimen of pulsed steroid therapy was intravenous administration of methylprednisolone (0.5 or 1.0 g/day for 3 consecutive days) followed by oral prednisolone at an initial dose of 0.6 mg/kg per day, with a gradual decrease in dosage over 1 year.

The clinical grade at the time of renal biopsy was determined according to the clinical grading criteria of the Japanese Society of Nephrology (JSN) [10] (Grade I, urinary protein (U-Prot) <0.5 g/day; Grade II, eGFR ≥60 ml/min and U-Prot ≥0.5 g/day; Grade III, eGFR <60 ml/min and U-Prot ≥0.5 g/day).

The histology sections were independently reviewed by two renal pathologists who were unaware of the patients’ clinical data. Histological severity was graded according to the histological grading criteria of the JSN [10] (Grade I, percentage of glomeruli with lesions <24.9 % of the total number of glomeruli; Grade II, percentage of affected glomeruli between 25 and 49.9 % of the total number of glomeruli; Grade III, percentage of affected glomeruli between 50 and 74.9 % of the total number of glomeruli; Grade IV, percentage of affected glomeruli >75 % of the total number of glomeruli). Glomerular lesions were classified as global sclerosis, segmental sclerosis and crescent formation. Interstitial cellular infiltration and tubular atrophy, as well as fibrosis, are mainly observed in Grade III or IV glomerular disease. The grades were appended with ‘A’ when there were acute lesions, e.g., cellular and fibrocellular crescents, and with ‘C’ when there were chronic lesions, e.g., global sclerosis, segmental sclerosis, and fibrous crescents.

The combination of clinical and histological grade according to the JSN [10] was also evaluated as low risk, medium risk, high risk and very high risk, including cases that had a low, medium, high and very high risk, respectively, of progression to ESKD within 5 years (Table 1).

**Table 1:** Combined clinical and histological grade according to the criteria of the Japanese Society of Nephrology

Clinical grade	Histological grade 1	Histological grade 2	Histological grade 3 and 4
1	Low risk	Medium risk	High risk
2	Medium risk	Medium risk	High risk
3	High risk	High risk	Very high risk

### Statistical analysis

Data are expressed as mean ± standard deviation (SD) or percentages. The results were analyzed using JMP^®^ 9.0.1 (SAS Institute Inc., NC, USA). Non-parametric variables were compared by using either the Mann–Whitney *U* test or the Kruskal–Wallis test. Categorical variables were compared by using either Fisher’s exact test or the chi-squared test. Comparison of the duration required to achieve the required outcome between the two groups was performed using the Kaplan–Meier method and the log-rank test. Multivariate Cox regression analysis was used to evaluate the factors favoring progression to the desired outcome in the two groups. *P* values of <0.05 were considered to be statistically significant in all the analyses.

## Results

### Baseline characteristics

Baseline characteristics in the two groups are summarized in Table 2. Age, follow-up period, duration from onset, BMI, sCr and eGFR were comparable between the two groups. In addition, the proportions of females and patients with hypertension, hematuria, those treated with RASI and those who underwent ST were also not different between the two groups (Table 2). However, the level of proteinuria in patients in the IgA-IgG group was significantly higher than that in the IgA group (1.1 ± 0.8 vs. 0.7 ± 0.6 g/day, *P* = 0.042).

**Table 2:** Comparison of baseline characteristics between IgA-IgG and IgA groups

	IgA-IgG group (*n* = 29)	IgA group (*n* = 28)	*P* value
Follow-up period (months)	36.2 ± 15.1	30.2 ± 15.4	0.131 M
Duration from onset (months)	27.1 ± 28.4	24.1 ± 33.9	0.290 M
Age (years)	36.2 ± 13.1	38.0 ± 13.3	0.511 M
Female gender, *n* (%)	15 (51.7 %)	14 (53.6 %)	0.896 F
BMI (kg/m^2^)	22.6 ± 3.5	22.9 ± 3.9	0.584 M
History of hypertension, *n* ( %)	9 (31.0 %)	14 (50.0 %)	0.149 F
Serum creatinine level (mg/dl)	1.0 ± 0.4	0.9 ± 0.3	0.226 M
eGFR (ml/min/1.73 mm^2^)	75.9 ± 26.0	69.2 ± 24.5	0.667 M
Proteinuria (g/day)	1.1 ± 0.8	0.7 ± 0.6	0.042* M
Hematuria, *n* ( %)			
1+	2 (6.9 %)	2 (7.1 %)	0.978 F
2+	10 (34.5 %)	8 (28.6 %)	0.631 F
3+	17 (58.6 %)	18 (64.3 %)	0.083 F
Use of RASI, *n* ( %)	26 (89.7 %)	22 (78.6 %)	0.251 F
Use of antiplatelet drugs, *n* (%)	28 (96.6 %)	26 (92.8 %)	0.532 F
Oral steroid therapy, *n* ( %)	22 (75.8 %)	20 (71.4 %)	0.704 F
Use of immunosuppressant drugs, *n* ( %)	1 (3.4 %)	0 (0 %)	0.329 F
Underwent steroid pulse therapy, *n* ( %)	19 (65.5 %)	18 (64.3 %)	0.922 F
Underwent tonsillectomy, *n* ( %)	20 (68.9 %)	19 (67.8 %)	0.928 F
Underwent ST, *n* ( %)	19 (65.5 %)	18 (64.3 %)	0.922 F

Data are shown as mean ± SD or number (percent). Mann–Whitney’s *U* test and Fisher’s exact test were used to compare baseline characteristics

*RASI* renin-angiotensin system inhibitor, *ST* steroid pulse therapy combined with tonsillectomy, *M* Mann–Whitney *U* test, *F* Fisher exact test

* *P* < 0.05

### Clinical and histological examinations

The distribution of clinical and histological grades in the two groups is shown in Table 3. With regard to the distribution of clinical grade (C-grade), the chi-squared test showed a significant difference between the two groups (*P* = 0.028). In the IgA-IgG group, patients with C-grade 2 were seen more frequently and those with C-grade 3 less frequently. In the IgA group, patients with C-grade 1 were seen more frequently and those with C-grade 3 less frequently.

**Table 3:** Clinical and histological grades in the two groups

	IgA-IgG group (*n* = 29)	IgA group (*n* = 28)	*P* value
Clinical grade			0.028*
Grade 1	6	14	
Grade 2	18	8	
Grade 3	5	6	
Histological grade			0.625
Grade 1 (total/A/AC/C)	17/2/12/3	19/2/15/2	
Grade 2 (total/A/AC/C)	11/1/8/2	5/1/4/0	
Grade 3 (total/A/AC/C)	1/0/1/0	3/0/3/0	
Grade 4 (total/A/AC/C)	0/0/0/0	1/0/1/0	
Clinical and histological grade			0.033*
Low	5	13	
Medium	19	6	
High	4	6	
Very high	1	3	

Data represent the number of patients. The chi-squared test was used to compare the distribution of clinical and histological grades

* *P* < 0.05

*A* acute lesion, *AC* acute and chronic lesion, *C* chronic lesion

In terms of histological grade (H-grade), H-grade 1 and 2 changes tended to be seen more frequently in all patients (Table 3). However, there were no significant differences between the two groups in the distribution of H-grades (Table 3).

Regarding the combination of clinical and histological grade, the distribution was significantly different between the two groups by the chi-squared test (*P* = 0.033). IgA-IgG group patients showed a higher tendency to medium risk. On the other hand, IgA group patients showed a higher tendency to low risk (Table 3).

### Immunostaining patterns

Immunostaining patterns of IgG and IgA in the present study are summarized in Tables 4 and 5. With regard to the immunofluorescence intensity of IgG, 12 patients were grade 1, 13 patients were grade 2, and 4 patients were grade 3 (Table 4). Of the 29 patients, mesangial and capillary wall IgG deposits were noted in 5 patients (17.2 %), whereas 24 patients (82.8 %) showed pure mesangial IgG deposits.

**Table 4:** Immunostaining patterns of IgG in IgA-IgG group

	IgA-IgG group patients (*n* = 29)
Immunofluorescence intensity	
Grade 1	12 (41.4 %)
Grade 2	13 (44.8 %)
Grade 3	4 (13.8 %)
Location of glomerular deposits	
Mesangial-only	24 (82.8 %)
Capillary wall + mesangial	5 (17.2 %)

Data represent the number (percent) of patients

**Table 5:** Immunostaining patterns of IgA in the two groups

	IgA-IgG group (*n* = 29)	IgA group (*n* = 28)	*P* value
Immunofluorescence intensity			0.029*
Grade 1	1 (3.4 %)	8 (28.6 %)	
Grade 2	16 (55.2 %)	16 (57.1 %)	
Grade 3	12 (41.4 %)	4 (14.3 %)	
Location of glomerular deposits			0.014*
Mesangial-only	12 (41.4 %)	25 (89.3 %)	
Capillary wall + mesangial	17 (58.6 %)	3 (10.7 %)	

Data represent the number (percent) of patients. The chi-squared test was used to compare the distribution of immunofluorescence intensity. Fisher’s exact test was used to compare the proportion of the locations of glomerular deposits

* *P* < 0.05

Regarding the immunofluorescence intensity of IgA, the distribution was significantly different between the two groups by the chi-squared test (*P* = 0.029). IgA-IgG group patients showed a higher tendency to grade 2 and 3. On the other hand, IgA group patients showed a higher tendency to grade 1 and 2 (Table 5). Concerning the location of glomerular IgA deposits, Fisher’s exact test showed a significant difference between the two groups (*P* = 0.014). In the IgA-IgG group, patients with mesangial and capillary wall deposits were seen more frequently; in the IgA group, patients with mesangial-only deposits were seen more frequently (Table 5).

### Cumulative probability of complete remission

**Fig. 3:**
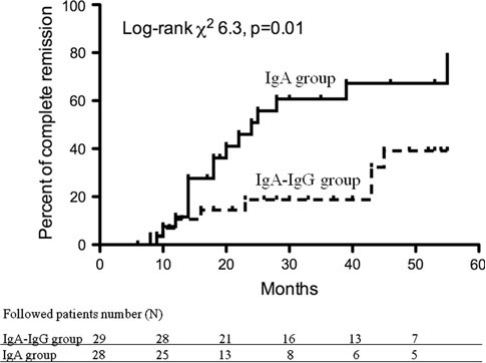
Cumulative probability of complete remission between IgA-IgG and IgA groups. Probability was analyzed by the log-rank test (*P* = 0.012)

During the median observation period of 33.3 months (range 6–55 months) in the 57 patients, 24 cases (42.1 %) had CR. The median observation period was 36.2 months (range 7–55 months) in the IgA-IgG group and 30.2 months (range 6–55 months) in the IgA group. The CR rate during the observation period was 27.6 % in the IgA-IgG group and 57.1 % in the IgA group. In addition, the median time to CR was 32.9 months in IgA-IgG group patients and 24.6 months in the IgA group. As shown in Fig. 3, the cumulative probability of CR was greater in the IgA group compared with that in the IgA-IgG group (log-rank test, *P* = 0.012).

### Multivariate analysis of factors that contribute to complete remission

The hazard ratio (HR) of possible factors related to CR and the 95 % confidence interval (CI) in the 57 patients with IgAN are shown in Table 6. In univariate analysis, IgG deposition reduced the HR for CR (HR 0.35; 95 % CI 0.14–0.82, *P* = 0.014), indicating that patients with IgG deposition were less likely to have CR. In multivariate analysis, female gender (HR 0.44; 95 % CI 0.16–0.93, *P* = 0.029) and greater amounts of proteinuria (HR 0.67; 95 % CI 0.43–1.01, *P* = 0.048) attenuated the HR for CR, indicating that females and patients with higher levels of protein in urine were less likely to have CR. In addition, multivariate analysis also demonstrated that IgG deposition reduced the HR for CR (HR 0.31; 95 % CI 0.12–0.77, *P* = 0.016). Therefore, both higher amounts of proteinuria and IgG deposition are risk factors for persistent urinary abnormalities. On the other hand, undergoing ST was a favorable factor for inducing CR (HR 3.47, 95 % CI 1.17–12.26, *P* = 0.023).

**Table 6:** Univariate and multivariate analysis of possible factors that contributed to complete remission in the 57 patients with IgAN

Variable	Univariate analysis			Multivariate analysis		
	HR	95 % CI	*P* value	HR	95 % CI	*P* value
Age (per 1 year of age)	1.06	(0.97–1.03)	0.833	1.04	(1.00–1.10)	0.172
Female gender (vs. male)	0.49	(0.21–1.12)	0.091	0.44	(0.16–0.93)	0.029*
Duration from onset (months)	0.99	(0.97–1.00)	0.158	0.99	(0.97–1.00)	0.157
IgG deposition (vs. absence)	0.35	(0.14–0.82)	0.014*	0.31	(0.12–0.77)	0.016*
History of hypertension (vs. absence)	0.87	(0.38–1.99)	0.772	0.57	(0.20–1.56)	0.278
Proteinuria (per 0.5 g/day)	0.75	(0.53–1.01)	0.504	0.67	(0.43–1.01)	0.048*
eGFR (per 10 ml/min)	1.04	(0.89–1.21)	0.644	1.03	(0.81–1.32)	0.821
Histological grade (per grade)	0.92	(0.47–1.62)	0.798	1.28	(0.64–2.34)	0.453
Use of RASI (vs. absence)	1.04	(0.30–6.53)	0.938	0.45	(0.09–3.42)	0.397
Underwent ST (vs. absence)	1.77	(0.74–4.92)	0.212	3.47	(1.17–12.26)	0.023*

*HR* hazard ratio, *CI* confidence interval, *RASI* renin-angiotensin system inhibitor, *ST* steroid pulse therapy combined with tonsillectomy

* *P* < 0.05

### Clinical and histological findings according to the immunostaining patterns of IgG

In IgA-IgG group patients, comparison of baseline characteristics and CR rate according to the patterns of IgG staining are summarized in Tables 7 and 8. With regard to immunofluorescence intensity, no significant differences in follow-up period, duration from onset, eGFR and proteinuria were seen between the three groups: grade 1, grade 2 and grade 3. In addition, the distribution of H-grade and the proportion of patients those who underwent ST and achieved CR were also not different between the three groups (Table 7).

**Table 7:** Comparison of baseline characteristics and complete remission rate according to the immunofluorescence intensity of IgG

	Grade 1 (*n* = 12)	Grade 2 (*n* = 13)	Grade 3 (*n* = 4)	*P* value
Follow-up period (months)	32.7 ± 14.6	38.5 ± 16.8	39.8 ± 11.1	0.528 K
Duration from onset (months)	21.8 ± 17.9	32.9 ± 37.6	19.0 ± 20.4	0.441 K
eGFR (ml/min/1.73 mm^2^)	69.7 ± 23.2	85.4 ± 27.5	64.0 ± 24.6	0.203 K
Proteinuria (g/day)	1.4 ± 0.8	0.9 ± 0.6	0.8 ± 0.5	0.173 K
Histological grade 1/2/3/4, *n*	5/6/1/0	8/5/0/0	4/0/0/0	0.270 C
Underwent ST, *n* ( %)	8 (66.7 %)	8 (61.5 %)	3 (75.0 %)	0.879 C
Achievement of CR, *n* ( %)	3 (25.0 %)	3 (23.1 %)	2 (50.0 %)	0.554 C

Data are shown as mean ± SD or number (percent). Kruskal–Wallis test and chi-squared test were used to compare baseline characteristics and proportion of complete remission between the three groups

*ST* steroid pulse therapy combined with tonsillectomy, *CR* complete remission, *K* Kruskal–Walls test, *C* chi-squared test

**Table 8:** Comparison of baseline characteristics and complete remission rate according to the location of glomerular IgG deposits

	Capillary wall + mesangial (*n* = 5)	Mesangial-only (*n* = 24)	*P* value
Follow-up period (months)	28.8 ± 11.9	37.8 ± 15.4	0.213 M
Duration from onset (months)	17.6 ± 18.8	28.2 ± 30.1	0.414 M
eGFR (ml/min/1.73 mm^2^)	86.6 ± 37.9	73.7 ± 23.4	0.299 M
Proteinuria (g/day)	1.2 ± 0.8	0.6 ± 0.5	0.091 M
Histological grade 1/2/3/4, *n*	5/0/0/0	12/11/1/0	0.270 C
Underwent ST, *n* ( %)	3 (60.0 %)	16 (66.7 %)	0.816 F
Achievement of CR, *n* (%)	3 (60.0 %)	5 (20.8 %)	0.554 F

Data are shown as mean ± SD or number (percent). The Mann–Whitney *U* test, Fisher exact test and chi-squared test were used to compare baseline characteristics and proportion of complete remission between the two groups

*ST* steroid pulse therapy combined with tonsillectomy, *CR* complete remission, *M* Mann–Whitney *U* test, *F* Fisher exact test, *C* chi-squared test

In terms of the location of glomerular deposits, follow-up period and duration from onset tended to be longer in patients with mesangial-only deposits, eGFR was lower in patients with mesangial-only deposits, proteinuria was greater in patients with mesangial and capillary wall deposits, but none of the differences were significant (Table 8). In addition, the distribution of H-grade and proportion of patients treated with ST were comparable. Furthermore, the CR rate tended to be higher in patients with mesangial and capillary wall deposits, although the differences were not significant.

## Discussion

Berger [4] first reported the existence of a glomerulonephritis characterized by IgA and IgG deposits in the glomeruli. Since then, several synonyms of IgAN have been reported, such as nephropathy with mesangial IgA and IgG deposits, IgA glomerulonephritis, Berger’s disease and IgA-IgG nephropathy [2]. With regard to IgG deposition rates in patients with IgAN, Haas showed an IgG deposition rate of approximately 45 % [11]. In addition, Okada et al. showed an IgG deposition rate of 50 % in 111 Japanese patients (children and adults) with IgAN [12]. In the present study, we classified 29 patients out of 57 (51 %) as positive for IgG deposits. Therefore, this study also showed a similar IgG deposition rate.

Recent analysis of the glycosylation of IgA1 in patients with IgAN has provided new insights into the mechanisms underlying the formation of immune complexes and their deposition in the mesangium. Specifically, aberrant glycosylation of *O*-linked glycans in the hinge region of a fraction of IgA1 molecules is a key pathogenic factor contributing to the development of IgAN [13]. It is known that in patients with IgAN, circulating IgA1 with aberrant hinge region glycans bind to IgG or IgA1 with antiglycan specificity [13, 14]. In addition, Novak et al. showed that IgAN-circulating immune complexes (CIC) containing aberrantly glycosylated IgA1 bind to mesangial cells (MC) more efficiently than uncomplexed IgA, and that CIC from an IgAN patient bind to MC to a greater extent than do CIC from a healthy control patient [15]. Collectively, the terminal *N*-acetylgalactosamine moiety on the aberrantly glycosylated IgA1 is probably recognized by antiglycan antibodies (IgG and/or IgA1), leading to the formation of nephritogenic CIC that subsequently deposit in the glomerular mesangium to induce kidney injury [16]. Therefore, we assume that the immune complexes composed of aberrant IgA1, with galactose-deficient hinge-region *O*-glycans, and an antiglycan-specific IgG antibody may determine the presence or absence of mesangial IgG deposition and could be the mechanism underlying the progression of glomerulonephritis. Furthermore, it was reported that observation of IgG deposition is affected by the timing of renal biopsy [8, 15], and that IgG deposition is often observed in patients with a long interval between the onset of IgAN and biopsy [8]. In the present study, the period between the onset of proteinuria and the time of renal biopsy was not significantly different between the two groups, but further studies will be necessary to determine the relation between mesangial IgG deposition and duration of IgAN.

Next, we examined whether mesangial IgG deposition might predict clinical outcome in patients with IgAN. Several prognostic factors, such as elevated sCr, massive proteinuria, hypertension, glomerular or interstitial sclerosis and IgA deposition in the peripheral capillary wall were demonstrated as being significantly associated with poor clinical outcome in previous studies [17]. On the other hand, ST has been reported to improve the CR rate in patients with IgAN [18]. Furthermore, the Oxford classification of IgAN identified four histological features––mesangial cellularity, endocapillary proliferation, segmental sclerosis and tubular atrophy/interstitial fibrosis––as independent predictors of clinical outcome [19, 20], although this classification did not mention the significance of immunostains in the analysis. However, it was reported that mesangial co-deposition of IgG with IgA was a significant risk factor for CKD progression in patients with IgAN [21]. D’Amico critically analyzed 23 valid studies on IgAN published in the preceding two decades, and noted that co-deposition of IgG was an independent risk factor affecting renal outcome [17]. In addition, it was reported that mesangial IgG deposition was associated with greater inflammation in an animal model [22], and mesangial IgG deposition was recently shown to be associated with greater histological activity [23]. In the present study, we demonstrated that patients with mesangial IgG-IgA deposition had greater proteinuria, lower CR rate, more frequent capillary wall IgA deposits, and resistance to treatment. In the multivariate analysis, we demonstrated that IgG deposition was a risk factor for persistent urinary abnormalities. Therefore, we suggest that assessing mesangial IgG deposition would be useful in evaluating the degree of disease activity, and that mesangial IgG deposition may be a useful predictor for estimating the effect of treatment in IgAN patients.

In Japan, Hotta et al. [18] initially described ST, which then became a popular approach to treating IgAN. However, there have been few reports of similar studies to ours that took the effects of ST into consideration. This makes our study results even more valuable. In addition, most of the reports did not mention the intensity and location of glomerular IgG deposits in patients with IgAN. In the present study, a correlation between immunostaining pattern of IgG in IgAN and clinical parameters or histological grade has not been demonstrated. However, validation of these findings is required in other cohorts, particularly the significance of the location of glomerular IgG deposits as well as IgA deposits. It is recommended that the location and intensity of IgA and IgG staining is routinely included in the renal biopsy report.

Our study has several limitations. First, the number of enrolled patients was small and the follow-up duration was short. Second, this study mainly targeted less severe patients. In fact, the proportion of patients with histological grade 3 or 4 was only 8.7 %, and only two patients had advanced IgAN with sCr of >2.0 mg/dl. Third, in patients who underwent ST, treatment periods, total steroid doses and timing of tonsillectomy were not uniform among the subjects. Fourth, we used surrogate markers, i.e. remission of proteinuria and hematuria, to assess renal outcome. True endpoints, based on the renal survival rate after longer periods, and not merely the CR rate, need to be evaluated. Lastly, since this was a retrospective observational study, residual confounding by imperfectly measured or unknown confounders may still be present.

## Conclusion

Mesangial IgG co-deposition with IgA could be associated with increased clinical severity and resistance to remission of urinary abnormalities in patients with IgAN. Mesangial IgG deposition may, thus, be useful in predicting the clinical outcome of IgAN.

## References

[1] Donadio JV, Grande JP (2002). IgA nephropathy. N Engl J Med.

[2] Tomino Y, Sakai H (2003). Special study group (IgA nephropathy) on progressive glomerular disease. Clinical guidelines for immunoglobulin A (IgA) nephropathy in Japan, second version. Clin Exp Nephrol.

[3] Emancipator SN. IgA nephropathy and Henoch–Schönlein syndrome. In: Jennette JC, Olson JL, Schwartz MM, Silva FG, editors. Heptinstall’s pathology of the kidney. Philadelphia: Lippincott-Raven Publishers; 1998, p. 479–539.

[4] Berger J, Hinglais N (1968). Les depots intercapillaires d’IgA-IgG (intercapillary deposits of IgA-IgG). J Urol Nephrol.

[5] Conley ME, Cooper MD, Michael AF (1980). Selective deposition of immunoglobulin A1 in immunoglobulin A nephropathy, anaphylactoid purpura nephritis, and systemic lupus erythematosus. J Clin Invest.

[6] Julian BA, Tomana M, Novak J, Mestecky J (1999). Progress in the pathogenesis of IgA nephropathy. Adv Nephrol.

[7] Novak J, Julian BA, Tomana M, Mestecky J (2001). Progress in molecular and genetic studies of IgA nephropathy. J Clin Immunol.

[8] Kanemoto K, Tobita N, Anzai M, Matsumura C, Udagawa J, Kitamura H (2009). Multilateral investigation of IgA-IgG co-deposition in pediatric IgA nephropathy and Henoch–Schonlein purpura nephritis. Jpn J Pediatric Nephrol.

[9] Imai E, Horio M, Nitta K, Yamagata K, Iseki K, Tsukamoto Y, et al. Modification of the Modification of Diet in Renal Disease (MDRD) Study equation for Japan. Am J Kidney Dis. 2007; 50:927–37. 10.1053/j.ajkd.2007.09.004 18037093

[10] Matsuo S (2011). Clinical guides for immunoglobulin A (IgA) nephropathy in Japan, third version. Jpn J Nephrol.

[11] Haas M. IgA nephropathy and Henoch–Schönlein purpura nephritis. In: Jennette JC, Olson JL, Schwartz MM, Silva FG, editors. Heptinstall’s pathology of the kidney, 6th edn. Philadelphia: Lippincott Williams & Wilkins; 2006. p. 423–86.

[12] Okada K, Funai M, Morimoto Y, Kagami S, Yano I, Kawakami K (1990). IgA nephropathy in Japanese children and adults: a comparative study of clinicopathological features. Am J Nephrol.

[13] Suzuki H, Fan R, Zhang Z, Brown R, Hall S, Julian BA (2009). Aberrantly glycosylated IgA1 in IgA nephropathy patients is recognized by IgG antibodies with restricted heterogeneity. J Clin Invest.

[14] Tomana M, Novak J, Julian BA, Matousovic K, Konecny K, Mestecky J (1999). Circulating immune complexes in IgA nephropathy consist of IgA1 with galactose-deficient hinge region and antiglycan antibodies. J Clin Invest.

[15] Novak J, Tomana M, Matousovic K, Brown R, Hall S, Novak L (2005). IgA1-containing immune complexes in IgA nephropathy differentially affect proliferation of mesangial cells. Kidney Int.

[16] Kiryluk K, Moldoveanu Z, Sanders JT, Eison TM, Suzuki H, Julian BA (2011). Aberrant glycosylation of IgA1 is inherited in both pediatric IgA nephropathy and Henoch–Schönlein purpura nephritis. Kidney Int.

[17] D’Amico G. Natural history of idiopathic IgA nephropathy and factors predictive of disease outcome. Semin Nephrol. 2004; 24:179–96. 10.1016/j.semnephrol.2004.01.001 15156525

[18] Hotta O, Miyazaki M, Furuta T, Tomioka S, Chiba S, Horigome I (2001). Tonsillectomy and steroid pulse therapy significantly impact on clinical remission in patients with IgA nephropathy. Am J Kidney Dis.

[19] Cattran DC, Coppo R, Cook HT, Feehally J, Roberts ISD, Troyanov S (2009). The Oxford classification of IgA nephropathy: rationale, clinicopathological correlations, and classification. Kidney Int.

[20] Roberts ISD, Cook HT, Troyanov S, Alpers C, Amore A, Barratt J (2009). The Oxford classification of IgA nephropathy: pathology, definition, correlation, and reproducibility. Kidney Int.

[21] Nieuwhof C, Kruytzer M, Frederiks P (1998). Van Breda Vriesman PJ. Chronicity index and mesangial IgG deposition are risk factors for hypertension and renal failure in early IgA nephropathy. Am J Kidney Dis.

[22] Van Dixhoorn MG, Sato T, Muizert Y, van Gijlswijk-Janssen DJ, De Heer E, Daha MR (2000). Combined glomerular deposition of polymeric rat IgA and IgG aggravates renal inflammation. Kidney Int.

[23] Bellur S, Troyanov S, Cook HT (2011). Roberts ISD on behalf of a working group of the international IgA nephropathy network and the renal pathology society. Nephrol Dial Transpl.

